# Implementation, coverage and impact of a programmatic mass drug administration campaign for malaria in southern Mozambique

**DOI:** 10.1186/s12889-026-28353-6

**Published:** 2026-07-03

**Authors:** Maria Tusell, Laura Fuente-Soro, Arsénio Nhacolo, Helena Martí-Soler, Jacopo Vecchio, Amancio Nhangave, Khalid Bapu, Felimone Amone, Christina Riley, Orcídio Chivite, Nteruma Catorze, Valencia Novele, Wilson Simone, Ismail Chiposse, Alfa Moiane, Alberto Gomo, Odety Chissico, Mércia Dimene, Samira Sibindy, Baltazar Candrinho, Pedro Aide, Caterina Guinovart

**Affiliations:** 1 Barcelona Institute for Global Health (ISGlobal), Barcelona, Spain; 2 Facultat de Medicina i Ciències de la Salut, Universitat de Barcelona (UB), Barcelona, Spain; 3 Manhiça Health Research Centre, Maputo, Mozambique; 4 Gaza Provincial Directorate of Health, Xai-Xai, Mozambique; 5 Akros Research, Lusaka, Zambia; 6 Manjacaze District Directorate of Health, Manjacaze, Mozambique; 7 Zavala District Directorate of Health, Zavala, Mozambique; 8 National Malaria Control Program, Ministry of Health, Maputo, Mozambique; 9 National Institute of Health, Ministry of Health, Maputo, Mozambique

**Keywords:** Mass drug administration, Coverage, Impact evaluation, Malaria elimination, Implementation science, Mixed-methods, Controlled-interrupted time series, Mozambique

## Abstract

**Background:**

Between 2022 and 2023, the Mozambican National Malaria Control Programme developed a locally-tailored implementation strategy for programmatic mass drug administration (pMDA), which was piloted in Chidenguele (Manjacaze District), southern Mozambique. Two rounds of door-to-door pMDA with dihydroartemisinin-piperaquine were conducted, targeting 59,271 individuals in 14,818 households, according to administrative data. Satellite imagery was used to support household enumeration and field navigation.

**Methods:**

An evaluation of the pMDA was conducted. Coverage was assessed using programmatic data and a community household survey (*n* = 770 individuals). A quasi-experimental design, using neighboring areas not receiving pMDA as a comparison group, was used to evaluate the impact of the pMDA on clinical malaria incidence during the ensuing 22 months through a controlled interrupted time-series analysis of routine surveillance data, adjusting for covariates.

**Results:**

According to programmatic data, household availability coverage (households reached/target households) increased from 59.4% (8,796/14,818) in the first round to 94.3% (13,972/14,818) in the second, following optimization of the implementation strategy. Programmatic or contact coverage (individuals treated/target population) increased from 41.2% (24,437/59,271) to 69.7% (41,320/59,271). In the second round, 8% of the target population was not reached, 8.7% were absent during visits, 6.9% were ineligible, and 6.7% refused participation. The household survey showed similar coverages and estimated that 81.6% of respondents (628/770, 95% CI 78.6–84.2) were treated in at least one of the rounds. We found weak evidence of a larger decrease in malaria incidence immediately following the pMDA implementation in the pMDA group versus the comparison group (level change incidence risk ratio [IRR] = 0.66, *p* = 0.074), and no evidence of a difference in the malaria trend over time between the two groups (trend change IRR = 0.96, *p* = 0.104).

**Conclusions:**

Although more than 90% of target households and individuals were reached in the second round, achieving the recommended 80% programmatic coverage remained challenging. This target was reached only when considering participation in either of the two rounds, highlighting the importance of conducting multiple rounds. High coverage requires strong community engagement, household revisits and/or fixed points, and substantial human and logistical resources. Using satellite imagery and triangulating with census data allowed us to estimate denominators, though challenges remain in this process. We found weak evidence of an impact on malaria incidence immediately following the pMDA and no evidence of a difference in the incidence trend over time. Overall, pMDA can be implemented under programmatic conditions, but is resource-intensive and should be reserved for specific contexts alongside other core malaria interventions.

**Supplementary Information:**

The online version contains supplementary material available at https://doi.org/10.1186/s12889-026-28353-6.

## Background

Mozambique was the fifth country worldwide with the highest burden of malaria cases in 2024, accounting for 3.6% of global cases [1]. In 2022, the average prevalence of *Plasmodium falciparum* (*P. falciparum*) infection in children under five years of age was 32.3%, although transmission intensity in the country is very heterogeneous, ranging from 0.3% in Maputo province – which is targeted for elimination – to 54.7% in Nampula province [2]. The National Malaria Control Program (NMCP) aims to reduce the burden of malaria in the high transmission areas and sustain progress toward elimination in the south, and revert the increasing trend in malaria cases observed between 2019 and 2023 [3, 4].

In view of the stagnating progress in malaria control over the past decade, the World Health Organization (WHO) has highlighted the need for a significant expansion in the use of chemoprevention, which is a key component of the WHO-recommended package of interventions to reduce transmission and prevent morbidity and mortality, alongside vector control and diagnostic testing and treatment [5]. Chemoprevention consists of full therapeutic courses of antimalarial medicines administered regardless of infection status at predefined intervals to treat existing infections and prevent new ones [6]. When all population age groups in a defined geographical area are targeted, the intervention is known as mass drug administration (MDA), which has historically been an important component of malaria control and elimination strategies [7]. In the 2022 WHO’s updated chemoprevention guidelines, the use of MDA was recommended to reduce disease burden in moderate to high transmission settings and in emergency settings, as well as to reduce transmission of *P. falciparum* in very low to low transmission settings, alongside good coverage of case management, vector control and surveillance [8]. To achieve significant impact, MDA campaigns should achieve at least 80% coverage of the target population, which requires a high level of community engagement to enhance community participation, acceptance and adherence, as well as appropriate delivery strategies to reach all households (HHs) in the targeted area [9].

Several MDA campaigns have been implemented in Mozambique in recent years. Between 2015 and 2018 the Magude Project implemented MDA and intensified vector control (long-lasting insecticidal nets (LLINs) and indoor residual spraying (IRS)) in a low transmission area in the south of the country [10, 11]. MDA campaigns for burden reduction in Cabo Delgado Province, located in the north of the country and hit by conflict, have also been implemented by the NMCP since 2021.

Given that the updated WHO recommendations provide more flexibility to countries to adapt the implementation of MDA to their specific context [6], it was important to understand the contextual factors that would affect MDA coverage and design an implementation strategy adapted to the Mozambican context, as no guidelines for programmatic MDA (pMDA) (i.e., deployed as a routine activity implemented by the NMCP through the national health system) were available in the country. In this context, we aimed to develop, pilot and evaluate an implementation strategy and monitoring plan for pMDA in Mozambique. This article presents the coverage and impact evaluation of the pMDA campaign. Additional implementation outcomes are presented elsewhere [12].

## Methods

### Setting

The pMDA was piloted and evaluated in the administrative post of Chidenguele (Manjacaze District, Gaza Province). It is one of the five administrative posts in the district, a rural coastal area located in southern Mozambique (24° 04’ south, 33° 56’ east). In 2021, the estimated population in the administrative post as provided by the district health directorate (based on the 2017 national census) was 59,271 individuals living in 14,818 HHs (assuming 4 people per HH). The area has several lakes and ponds surrounded by sandy soils and coastal dunes [13]. 

Malaria transmission is perennial and low, with the peak season coinciding with the rainy season, which has historically been between November and March. Malaria case incidence in 2021 was 121 cases per 1000, according to routine surveillance data reported in the national health management information system (HMIS). Chidenguele has seven rural health facilities (HFs) and nine community health workers (CHWs). Monthly malaria case counts are reported by each HF and are electronically submitted to the HMIS using the District Health Information Software 2 (DHIS2) (https://dhis2.org*)* at the district level. To improve the accuracy of the reported data, routine data quality assessments have been implemented since 2019 [14].

Malaria control in the area includes case management, intermittent preventive treatment for pregnant women with sulfadoxine-pyrimethamine, LLINs distributed through door-to-door campaigns conducted every 3–4 years (most recent ones conducted in November/December of 2020 and November of 2023) and regularly distributed through antenatal clinics, and IRS since 2006 (most recent cycles implemented between October of 2022 and March of 2023 and between October of 2023 and February of 2024).

### The pMDA implementation strategy

A participatory process led by the NMCP, the Manhiça Health Research Centre (CISM) and the Barcelona Institute for Global Health (ISGlobal), with the participation of other country stakeholders, was conducted to develop the implementation strategy. The pMDA implementation was led by the NMCP and the province and district health authorities, with CISM’s and ISGlobal’s support. Two rounds of pMDA were implemented during the peak transmission season: the first round took place in December 2022 (pMDA1, duration 12 days) and the second round five weeks later, between January and February of 2023 (pMDA2, duration 15 days). A total of 28 coordinators, 144 fieldworkers, 72 local community guides and 9 supervisors were involved in the implementation. Coordinators and supervisors were appointed by the NMCP at the national and provincial levels, while fieldworkers and community guides were selected at the district level by the coordinators. The main delivery mode was through door-to-door visits, complemented by one or two days of distribution in pre-established fixed points (markets, churches, schools, and other busy venues) that were previously announced by the community engagement teams. All individuals older than six months living in or visiting Chidenguele were eligible to participate in the campaign. Teams of two fieldworkers and a local community guide visited the HHs in Chidenguele, offering treatment to all eligible and consenting individuals. The treatment consisted of a full, age-adjusted, three-day therapeutic course of dihydroartemisinin-piperaquine (DHAP, Eurartesim^®^, Alfasigma, Italy). To maximize the chances of finding all individuals, HHs were visited up to three times. Exclusion criteria included being under six months of age, pregnancy (any trimester), reported heart conditions, having taken antimalarials in the previous 15 days, having taken contraindicated medicines in the previous seven days, current fever or history of fever within the past 24 h, and being seriously ill. For the last two cases, individuals were referred to a HF. Directly observed treatment (DOT) was performed by the fieldworkers during the visit only for the first dose. Fieldworkers explained how and when to take the second and third doses, which were left with the participants together with a card with information about the dosages for each member of the HH. For pMDA1, a HH questionnaire and one individual questionnaire for each HH member, including absent and non-participating individuals, were completed using a standardized electronic form filled out in DHIS2 (S1 Appendix). For pMDA2, to reduce the time spent in each HH, data collection was changed to only one HH-level questionnaire with the aggregated number of individuals present, absent, treated and refusing to participate, by age. The HH geo-location was recorded in both rounds (S2 Appendix). These programmatic data were submitted daily to the DHIS2 server to feed the dashboards used to monitor the implementation of the campaign. Further information on the pMDA operationalization and implementation can be found in the supplementary information (S3 Appendix).

Monitoring of adverse events (AEs) was done through the routine national pharmacovigilance system. Both the pMDA teams and the area’s HFs’ staff were trained to report any AEs in pMDA participants, and all participating individuals were advised to seek care at the HF in case of any AE. Moreover, the most frequent AEs were listed on the card that was given to the families so they could also recognise and report potential AEs.

A community engagement campaign in collaboration with World Vision was implemented one week before each pMDA round and throughout the campaign. Engagement meetings with district authorities and community and religious leaders were done, as well as mobilization in venues with a high concentration of people such as churches and markets. Before and during the pMDA, community mobilizers conducted door-to-door sensitization activities in all neighborhoods and communities, emphasizing the benefits of participation and revisiting areas or HHs where refusal rates were high. 

#### Satellite imagery maps

Satellite imagery (SI) maps were used throughout the campaign to aid planning, implementation and progress monitoring. Prior to the implementation, all HHs in the target area were enumerated and mapped using Reveal^®^ (Akros, https://revealprecision.com/*).* The target area was divided into equally sized delivery areas, which were later allocated to the fieldworker teams. During the implementation phase, the maps were transferred into an in-field application, which, combined with Global Positioning System navigation, guided the teams in the field to their assigned areas. When the fieldworkers reached each mapped structure, they were able to validate whether it was an eligible HH for the pMDA or not. If they found HHs that did not appear on the map, they were able to add them, providing an accurate HH target denominator. Fieldworkers marked eligible HHs on the map based on their visitation status as “completed”, “not or partially completed”, or “refused to participate/long-term absence”. This information was additionally transferred onto dashboards for real-time monitoring of the campaign’s progress. After field verification, the final number of eligible HHs identified in the administrative post of Chidenguele through SI was 14,045. Based on this number of HHs, the estimated population according to SI estimates was 57,304. This calculation assumes an average HH size of 4.08 members, derived from the data collected during the second round. 

#### Optimization of the implementation strategy

To improve the operational performance of pMDA2, the implementation strategy was optimized based on the lessons learnt during pMDA1. The delivery strategy was switched from a “scanning” strategy where all teams of fieldworkers were allocated to the same areas (i.e., villages) to achieve the maximum coverage in one day and then move on to the neighboring village, to a “decentralized” strategy where each team was allocated to a set of villages for the entire pMDA campaign to facilitate recovery visits for absent individuals. Community engagement was strengthened, increasing the number of community mobilizers and enhancing the involvement of the community leaders in the mobilization activities. The number of fieldworkers was also increased, and the duration of the campaign was increased from 12 to 15 days to accommodate some additional time for revisits and/or fixed points. Data collection was switched from individual to aggregated at the HH-level to decrease the time spent in the HHs, and the daily number of HHs that each team had to reach was decreased. An additional day of distribution points at the end of the campaign was also incorporated in an effort to reach as many individuals as possible. Further information about the operationalization and implementation strategy of the pMDA can be found in the S3 Appendix.

### Evaluation design

Coverage was assessed using the programmatic data collected during the pMDA implementation as well as through a community-based cross-sectional HH survey (HH survey). Additional implementation outcomes were evaluated following a mixed-methods approach and are reported elsewhere [12].

As a secondary objective, a quasi-experimental design was used to evaluate the impact of the pMDA on clinical malaria incidence using routine surveillance data through a controlled interrupted time-series (cITS) analysis, where selected Health Facility Catchment Areas (HFCAs) from the neighboring district of Zavala (Inhambane Province) were used as a comparison group. 

#### Community-based cross-sectional household survey.

A HH survey was conducted three weeks after the end of pMDA2 in a sample of 770 randomly selected individuals using multistage cluster sampling. Using the HH Reveal map from pMDA2, areas with the same number of HHs were created, from which 154 were randomly selected. Within each of the selected areas, 5 HHs were randomly selected, and within each HH one individual was randomly chosen. All HHs and individuals were eligible to be selected, including those not visited during the pMDA. To maximize the chances of finding the selected individuals, HHs were visited up to three times. If the selected individual refused to participate in the survey or was absent after three visits, a different HH member was randomly selected. If the individual was too young to respond independently, a parent was asked to answer on his/her behalf. However, participants were encouraged to respond for themselves whenever possible, without influence from others. The sample size was estimated assuming the different outcomes would have a prevalence of 50% (to provide the most conservative estimate), with a confidence level of 95%, a margin of error of 5% and a design effect of 2. After obtaining informed consent, a standardized electronic questionnaire using Open Data Kit (ODK, https://getodk.org/*)* was filled out for each participant by trained fieldworkers who were not involved in the pMDA implementation. Data were transferred into Research Electronic Data Capture (REDCap, https://www.project-redcap.org/*)* tools hosted at CISM’s server [15]. Data were collected on sociodemographics, participation in the pMDA, acceptability, and AEs.

### Statistical analysis and implementation outcomes

#### Descriptive analysis of implementation outcomes.

Implementation outcomes are presented as proportions calculated from programmatic data (involving the total population) and data collected during the HH survey. For the latter, 95% confidence intervals (CI) were computed. Statistical analyses were performed using Stata, version 18.0 [16].

##### Definition of target population and outcomes

The pMDA target population was the entire population of the administrative post of Chidenguele that slept in a HH for at least three nights/week during the implementation period, including visitors and regardless of whether they were present or not at the moment of the visit. Two different data sources were used to estimate HH and population denominators: census data and SI estimates. S1 Table presents the definitions used for the coverage outcomes, both at the HH- and individual-level and by data source. 

#### Quasi-experimental impact evaluation

A cITS analysis with a comparison group was conducted to evaluate the impact of the pMDA campaign on malaria incidence. We assessed whether the pre-intervention to post-intervention change in malaria incidence just after the implementation of the pMDA was different in the intervention group versus the comparison group (level or intercept change) and whether the change in the incidence trend over the 22 months following the intervention (compared to the two years pre-intervention) was different in the intervention group versus the comparison group (trend or slope change) [17].

##### Statistical methods

We used a generalized estimating equations model with negative binomial distribution and an autoregressive correlation structure, adjusted for covariates. The interruption, indicating when the pMDA was implemented, was included for December 2022 to February 2023. The pre-intervention period was from November 2020 to November 2022 and the post-intervention period from March 2023 to December 2024. Statistical analyses were performed using R, version 4.4.2 [18]. Further details of the model structure and description are presented in S4 Appendix.

##### Dependent variable (outcome)

The monthly number of confirmed malaria cases detected by microscopy or RDT at the HF or by CHWs was obtained from the national DHIS2 routine surveillance system for all intervention and comparison HFs. To ensure data quality, selected HFs in Chidenguele were visited to perform data quality audits, and data were corrected in case of any reporting errors. The HFCA population denominators to calculate incidences were provided by the district authorities and were included as offset in the model.

##### Model covariates

The following covariates were considered to be potentially associated with the dependent variable and were tested in the model: monthly number of outpatient visits to the HFs, number of CHWs, coverage of LLINs and IRS, and environmental factors with a 1- and 2-month lag (rainfall, temperature and enhanced vegetation index). The number of outpatient visits to the HFs was obtained from DHIS2. Coverage of LLINs and IRS were estimated based on data provided by the district authorities. The number of LLINs distributed during universal coverage campaigns was only available by locality and estimates at the HFCA-level were assumed to be proportional to the HFCA population. To estimate the number of HHs that received IRS, the total number of structures or huts reported to have been sprayed during the campaign was divided by 2.1 (based on data from the pMDA, each HH has an average of 2.1 structures), and reported each month cumulatively. To estimate the number of HHs that received IRS by HFCA, the number of HHs sprayed by locality was weighted based on the population density in each HFCA. The target number of HHs to receive IRS per HFCA was estimated by dividing the population at each HFCA by 4 (4 members/HH is what the district authorities consider when planning IRS). IRS waning effectiveness over time was accounted for by applying a decay function to the coverage, to capture the loss in insecticide residual efficacy starting in the month when IRS was conducted, and assuming that after 12 months the coverage was zero [19]. Enhanced vegetation index data were obtained from the moderate resolution imaging spectroradiometer (MODIS) (MODISTools R package) [20] and rainfall and temperature data were obtained from the Visual Crossing database [21]. The final model included the following covariates: monthly number of outpatient visits to the HFs, temperature (with a 1- and 2-month lag), rainfall (with a 1- and 2-month lag), and enhanced vegetation index (with a 1- and 2-month lag) (S3 Fig., S4 Fig.). These final covariates and the autoregressive correlation structure matrix were selected using the significance level, the quasi-likelihood under the independence model criterion (QIC), and the residuals [22].

## Results

### Coverage

#### Household-level

**Fig. 1:**
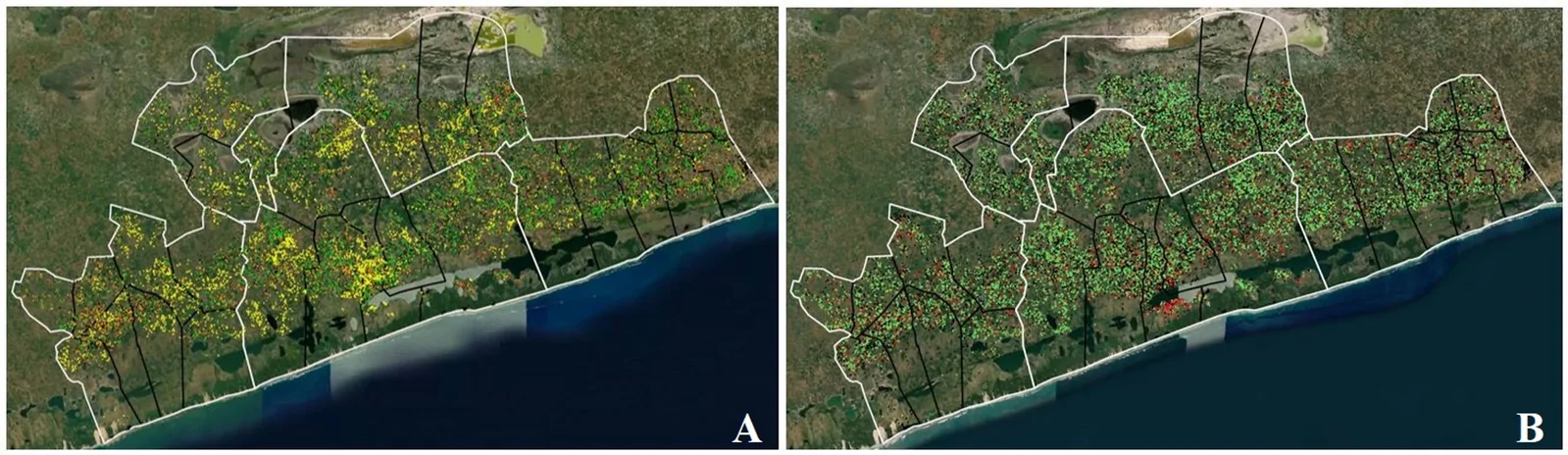
Map of HHs in Chidenguele with pMDA1 (**A**) and pMDA2 (**B**) HH status. Map of all HHs enumerated with SI: not visited (yellow), pMDA completed (green), pMDA partially completed (some HH members were absent) or empty HH (orange), pMDA refused or the HH members were absent at the moment of the revisits (red), and building not eligible (i.e., not a HH or abandoned HH) (black)

Availability coverage using census data denominators increased from 59.4% (8,796/14,818) in the first round to 94.3% (13,972/14,818) in the second. When accounting only for those HHs that were visited (where the pMDA team was able to talk to someone, including HHs that accepted to participate and HHs that refused), accessibility coverage was 43.0% (6,373/14,818) in the first round and increased to 80.5% (11,931/14,818) in the second. These coverages increased slightly when using the SI population denominators, reaching a 99.5% (13,972/14,045) availability in the second round (Table 1; Fig. 1, S1 Fig.).

**Table 1:** Summary of pMDA coverage by pMDA round and source of data

pMDA round	pMDA1			pMDA2		
Source of data	Census data % (*n*/*N*)	SI estimates % (*n*/*N*)	Cross-sectional household survey % (*n*/*N*, 95% CI)	Census data % (*n*/*N*)	SI estimates % (*n*/*N*)	Cross-sectional household survey % (*n*/*N*, 95% CI)
Household-level						
Availability coverage	59.4 (8,796/14,818)	62.6 (8,796/14,045)	64.5* (497/770, 61.1–67.9)	94.3 (13,972/14,818)	99.5 (13,972/14,045)	89.1* (686/770, 86.7–91.2)
Accessibility coverage	43.0 (6,373/14,818)	45.4 (6,373/14,045)	-	80.5 (11,931/14,818)	84.9 (11,931/14,045)	-
Empty households	27.5 (2,423/8,796)		-	14.6 (2,041/13,972)		-
Acceptability coverage	93.5 (5,959/6,373)		-	91.9 (10,966/11,931)		-
Participation refusal	6.5 (414/6,373)^1^		-	8.1 (965/11,931)^2^		-
Individual-level						
Availability coverage	63.4 (37,556/59,271)	65.5 (37,556/57,304)	64.8* (499/770, 61.3–68.2)^3^	92.0 (54,550/59,271)	95.2 (54,550/57,304)	89.1* (686/770, 86.7–91.2)
Accessibility coverage	45.4 (26,923/59,271)	46.9 (26,923/57,304)	60.9* (469/770, 57.4–64.4)^4^	83.3 (49,378/59,271)	86.2 (49,378/57,304)	81.9* (631/770, 79.0-84.6)
Programmatic or contact coverage	41.2 (24,437/59,271)	42.6 (24,437/57,304)	54.2* (417/770, 50.6–57.7)	69.7 (41,320/59,271)	72.1 (41,320/57,304)	73.9* (569/770, 70.6–77.0)
Effective coverage	65.1 (24,437/37,556)		83.6* (417/499, 80.0-86.7)	75.7 (41,320/54,550)		82.9* (569/686, 79.9–85.7)
Operational coverage	90.8 (24,437/26,923)		88.9* (417/469, 85.7–91.6)	83.7 (41,320/49,378)		90.2* (569/631, 87.6–92.4)
Treatment coverage among individuals that accept	92.2 (24,437/26,501)		92.3 (417/452, 89.4–94.5)	91.0 (41,320/45,402)		93.7 (569/607, 91.5–95.5)
Treatment coverage among eligible individuals	99.1 (24,437/24,648)		-	100 (41,320/41,320)		-
Absences among reached	28.3 (10,633/37,556)		6.0 (30/499, 4.1–8.5)	9.5 (5,172/54,550)		8.0 (55/686, 6.1–10.3)
Ineligibility among individuals that accept	6.4 (1,691/26,501)		-	9.0 (4,082/45,402)		-
Acceptability coverage among visited	98.4 (26,501/26,923)		96.4* (452/469, 94.3–97.9)	91.9 (45,402/49,378)		96.2* (607/631, 94.4–97.5)
Participation refusal among visited	1.6 (422/26,923)^5^		3.6* (17/469, 2.1–5.7)	8.1 (3,976/49,378)		3.8* (24/631, 2.5–5.6)^6^

*Abbreviations*: *CI* confidence interval

^1^Includes 4 HHs that were visited where participation is unknown

^2^Includes 15 HHs that were visited where participation is unknown

^3^Includes 2 individuals whose HH was not reached and were reached at the distribution point

^4^Includes 14 individuals that were visited at the distribution point

^5^Includes 7 individuals that were visited whose participation is unknown

^6^Includes 4 individuals that did not accept to participate due to reasons other than refusal and 1 individual whose participation is unknown

* Some coverage estimates from the cross-sectional household survey are also reported in Fuente-Soro et al. [12]

Data from the HH survey showed higher reported availability coverage for both rounds, with 64.5% (497/770, 95% CI 61.1–67.9) in the first round and 89.1% (686/770, 95% CI 86.7–91.2) in the second.

The proportion of empty HHs was 27.5% (2,423/8,796) and 14.6% (2,041/13,972) in pMDA1 and pMDA2, respectively. In terms of HH participation, participation refusal was 6.5% (414/6,373) in the first round and 8.1% (965/11,931) in the second (Table 1). The main reasons for refusal in both rounds were: not considering taking the medication necessary or beneficial (32.9%, 136/414 in pMDA1 and 26.3%, 254/965 in pMDA2) and fear of AEs (13%, 54/414 in pMDA1 and 18%, 174/965 in pMDA2). For the second round, the third main reason for refusal was because someone in the HH had suffered from AEs in the first round (16.1%, 155/965). 

#### Individual-level

Availability coverage using the census target population was 63.4% (37,556/59,271) in pMDA1 and 92% (54,550/59,271) in pMDA2. When using SI denominators, availability coverage increased to 65.5% (37,556/57,304) and 95.2% (54,550/57,304) in the first and second rounds, respectively. Data from the HH survey showed a similar availability coverage for pMDA1 (64.8%, 499/770, 95% CI 61.3–68.2) and a lower coverage for pMDA2 (89.1%, 686/770, 95% CI 86.7–91.2). Using the census denominators, accessibility coverage increased from 45.4% (26,923/59,271) in pMDA1 to 83.3% (49,378/59,271) in pMDA2, whereas according to HH survey data, it was 60.9% (469/770, 95% CI 57.4–64.4), and 81.9% (631/770, 95% CI 79.0-84.6), respectively. Out of all individuals visited in the second round, 10.2% (5,031/49,378) were located during a revisit and 8.3% were located at a fixed point.

**Fig. 2:**
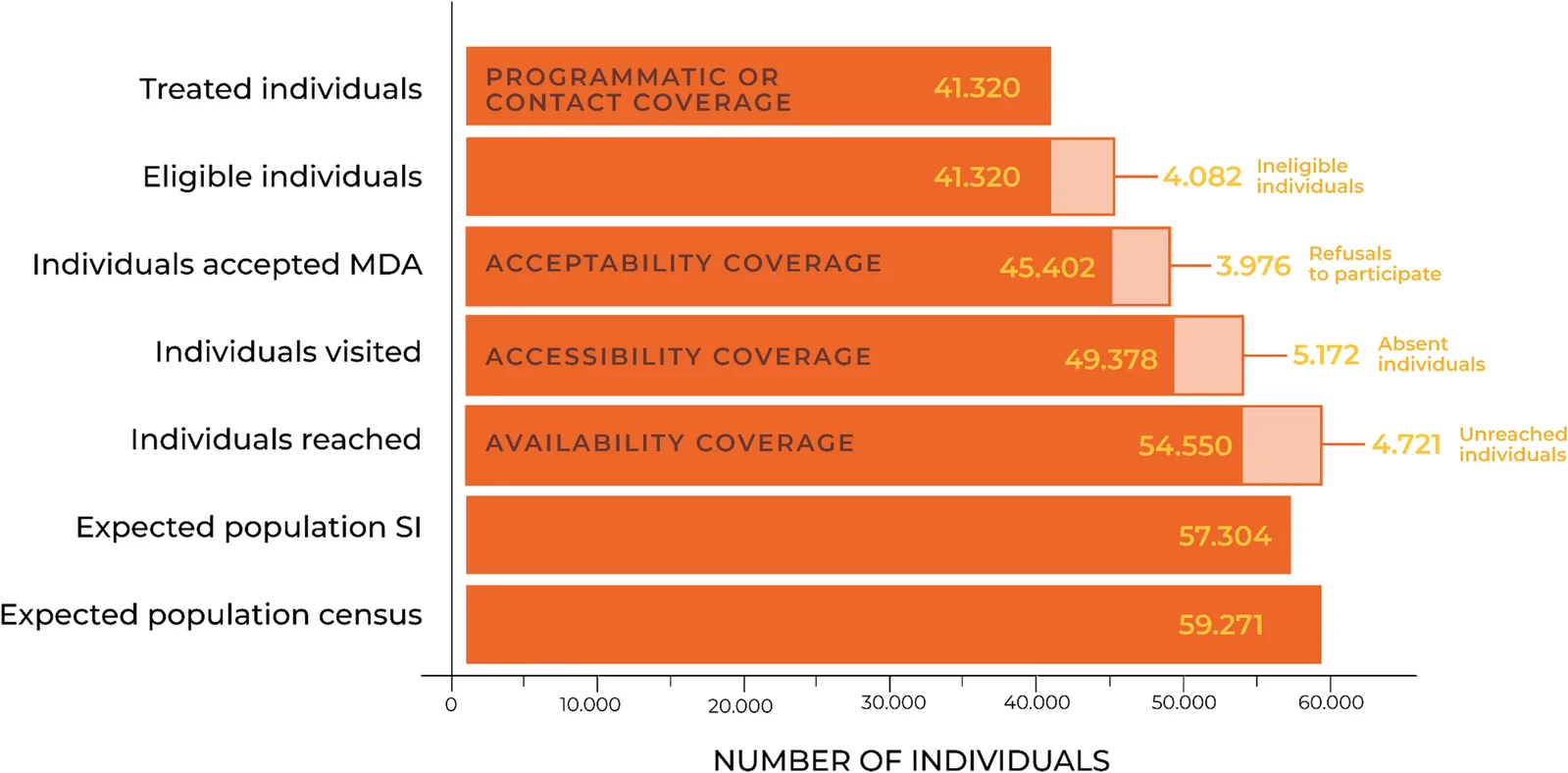
pMDA2 coverage cascade at the individual-level. MDA: mass drug administration; SI: satellite imagery. Expected population from census is based on the 2017 national census; expected population from SI was estimated assuming 4.08 individuals per HH

Programmatic or contact coverage using census data increased from 41.2% (24,437/59,271) in the first round to 69.7% (41,320/59,271) in the second. When using SI denominators, in the second round it was 72.1% (41,320/57,304). The distribution of the population treated by age and sex in pMDA1 and by age in pMDA2 is presented in S2 Table. Programmatic or contact coverage using data from the HH survey was slightly higher for both rounds: 54.2% (417/770, 95% CI 50.6–57.7) in pMDA1 and 73.9% (569/770, 95% CI 70.6–77.0) in pMDA2. The proportion of survey respondents that reported being treated in both rounds was 46.5% (358/770, 95% CI 42.9–50.1), whereas 81.6% (628/770, 95% CI 78.6–84.2) reported being treated in at least one of the rounds. Effective coverage was 65.1% (24,437/37,556) and 75.7% (41,320/54,550) in the first and second rounds, respectively, and among all individuals visited, operational coverage was 90.8% (24,437/26,923) in pMDA1 and 83.7% (41,320/49,378) in pMDA2 (Table 1; Fig. 2, S2 Fig.).

Although availability and accessibility coverages improved in the second round, the recommended target of 80% programmatic coverage was not achieved. Based on census data, 8% (4,721/59,271) of the target population were not reached by the pMDA teams, while an additional 8.7% (5,172/59,271) were absent during HH visits. Furthermore, 6.9% (4,082/59,271) were excluded due to ineligibility, and 6.7% (3,976/59,271) refused to participate. Despite trying to revisit all HHs that were empty at the moment of the first visit or with absent HH members, 9.5% (5,172/54,550) of individuals reached in pMDA2 were still absent (Table 1). Moreover, ineligibility among individuals that accepted was 6.4% (1,691/26,501) in pMDA1 and 9.0% (4,082/45,402) in pMDA2 (Table 1). Most common exclusion criteria in pMDA1 were reporting heart conditions (17.6% of all exclusion criteria; 298/1,691), having taken antimalarials in the previous 15 days (15.6%; 263/1,691), being pregnant (14.3%; 241/1,691), having taken contraindicated medicines in the previous seven days (14.2%; 240/1,691), age below six months (13.1%; 222/1,691), and fever or history of fever in the previous 24 h (8.2%; 138/1,691). Due to aggregate data collection, exclusion criteria were not registered in pMDA2. Participation refusal among those visited was 1.6% (422/26,923) in the first round and 8.1% (3,976/49,378) in the second (Table 1). Similar to the reasons for refusal at the HH-level, in pMDA1 the most common reasons for refusal at the individual-level were not considering the medication necessary or beneficial (26.8%; 113/422) and fear of AEs (10.2%; 43/422). Reasons for refusal at the individual-level were not registered for pMDA2.

### Safety outcomes 

There were 76 AEs recorded through the passive pharmacovigilance system, 35 in pMDA1 and 41 in pMDA2. The most common AE was headache (32.9%; 25/76), followed by abdominal pain (23.7%; 18/76), fever (11.8%; 9/76) and pruritus (10.5%; 8/75). One sudden unexplained death of an 85-year-old female was reported during the second round of the pMDA, one day after having completed the treatment with DHAP (three tablets of the 320/40 mg formulation per day for three days). A detailed investigation was conducted by the health authorities, including a verbal autopsy, which revealed that she had no history of any medical conditions or recent hospital admissions. She complained of lower abdominal pain after taking the medication and sought care at the HF, where no specific diagnosis was made. She went back home and died the next day. The verbal autopsy was inconclusive.

According to the data from the HH survey, 26.4% (150/569, 95% CI 22.8-30.2) of the participants that took the medication reported suffering one or more AEs, 62% felt them on the day they took the first dose of DHAP or one day after, and 38% felt them two, three or more days after taking the first dose. 96.7% reported having completed the three-day-course medication regimen despite suffering AEs (145/150, 95% CI 92.4-98.9) and 26% (39/150, 95% CI 19.2-33.8) reported having sought care at a HF. Out of the 231 AEs reported by the participants, the most common were dizziness (31.6%; 73/231, 95% CI 25.7-38.0), headache (18.2%; 42/231, 95% CI 13.4-23.8), malaise (17.7%; 41/231, 95% CI 13.0-23.3), and arthralgia (12.1%; 28/231, 95% CI 8.2-17.0). 

### Impact evaluation 

**Fig. 3:**
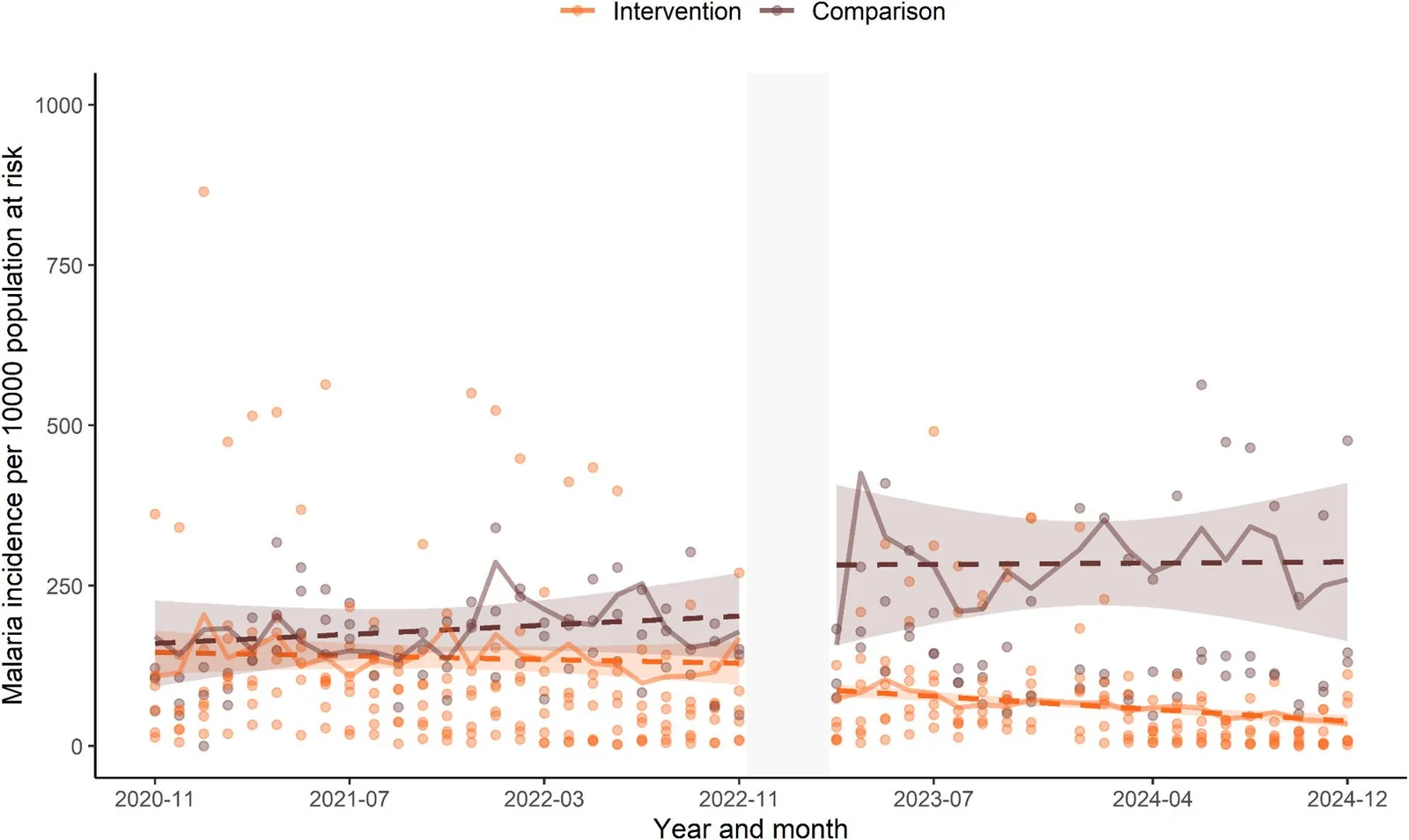
Monthly incidence of passively-detected malaria cases in intervention and comparison groups. Observed (dots) and fitted (line) monthly malaria incidence by health facility catchment area and intervention group and fitted trend of monthly malaria incidence by intervention group (dashed line). The vertical gray bar indicates the pMDA implementation period (December 2022-February 2023)

We found weak evidence against the null hypothesis that there is no difference in the incidence of malaria level change immediately after the pMDA in the pMDA group versus the comparison group (incidence risk ratio [IRR]=0.656, 95% CI 0.413-1.042, p=0.074). Moreover, we found no evidence against the null hypothesis that there is no difference in the incidence trend change over time in the pMDA group versus the comparison group (IRR=0.956, 95% CI 0.905-1.009, p=0.104) (Table 2). Figure 3 shows the monthly incidence of passively detected malaria cases by HFCA (observed and fitted) and the fitted trend of monthly malaria incidence by intervention group.

**Table 2:** Results of cITS regression analysis

	IRR	95% CI	*P*-value
Intervention components:			
Level in comparison group pre-intervention (β_0_)	0.008	0.005–0.011	0.000
Trend in comparison group pre-intervention (β_1_)	0.991	0.978–1.003	0.146
Level in comparison group post-intervention (β_2_)	1.363	0.721–2.578	0.340
Change in pre-post trend in comparison group (β_3_)	1.009	0.992–1.027	0.304
Difference in level between comparison and intervention groups pre-intervention (β_4_)	0.900	0.437–1.855	0.775
Difference in trend between comparison and intervention groups pre-intervention (β_5_)	1.006	0.980–1.032	0.672
Difference in post-intervention vs. pre-intervention level between comparison and intervention groups (β_6_) (level change)	0.656	0.413–1.042	0.074
Difference in post-intervention vs. pre-intervention trend between comparison and intervention groups (β_7_) (trend change)	0.956	0.905–1.009	0.104
Model covariates:			
Monthly number of outpatient visits to HF	1.000	1.0002–1.0005	0.000
Coverage of IRS	1.451	1.100-1.914	0.008
Rainfall (1-month lag)	0.957	0.944–0.971	0.000
Rainfall (2-months lag)	1.021	1.008–1.034	0.002
Temperature (1-month lag)	1.142	1.070–1.220	0.000
Temperature (2-months lag)	1.011	0.964–1.059	0.659
Enhanced vegetation index (1-month lag)	1.000	1.000-1.001	0.002
Enhanced vegetation index (2-months lag)	0.999	0.9995–0.9998	0.000

*CI* confidence interval, *cITS* controlled interrupted time-series, *HF* health facility, *HFCA* health facility catchment area, *IRR* incidence risk ratio, *LLIN* long-lasting insecticidal net, *pMDA* programmatic mass drug administration

## Discussion

We evaluated the coverage and impact of a pMDA implemented in Chidenguele, Manjacaze District, southern Mozambique. The implementation strategy and monitoring plan, co-created by the NMCP and partners, were successfully implemented under programmatic conditions. However, despite reaching more than 90% of target HHs and individuals during the second round, the recommended 80% programmatic coverage was not achieved, consistent with other MDAs in low-transmission settings, both programmatic [23] and in the context of trials [11, 24–27]. Key barriers limiting coverage included HH not reached by pMDA delivery teams, absenteeism during visits, exclusions due to ineligibility (e.g., heart conditions, recent antimalarial use, pregnancy, contraindicated medications), and refusals. Despite conducting up to two revisits per HH to increase the chances of finding absent individuals, over 9% of those reached (i.e., registered) in pMDA2 were not present during any of the HH visits. Using administrative census data, this corresponds to 8.7% of the 59,271 individuals in the target population.

Additionally, 6.4% of the individuals who accepted to participate in pMDA1 and 8.0% in pMDA2 were not eligible to receive the medication. Refusals in pMDA2 accounted for 6.7% of the target population. With this proportion of unreached, absences, exclusions, and refusals, achieving the recommended 80% programmatic coverage (which uses the total target population as the denominator) becomes difficult and leaves limited margin for other unforeseen challenges. However, when considering participation in either of the two rounds, results from the HH survey showed that over 80% of the target population received treatment, stressing the value of conducting multiple rounds to maximize coverage.

Consistent with our findings, absence from home was one of the main barriers to high coverage according to a systematic review looking at the contextual factors influencing MDA [28] and absenteeism of HH heads was also identified as the main barrier for the success of the MDA campaign conducted within the Magude Project [29]. Strong community engagement, household revisits, and revisiting some exclusion criteria could help improve coverage.

Registering HH members who were absent at the time of the pMDA visit was another challenge. Fieldworker training emphasized the importance of registering all members of the HH, including those who were not present at the moment of the visit, to ensure accurate estimation of denominators. To avoid duplication of individuals, data had to be edited to correctly account for individuals found in a revisit who had been registered as absent on the first visit. Despite these challenges, revisits significantly contributed to increasing coverage, given that 10% of the individuals visited in the second round were found during a revisit. Furthermore, as fieldworkers only performed DOT for the first dose, no programmatic information on adherence was available. A potential option for monitoring adherence to the second and third doses in the context of pMDAs could be asking supervisors to visit a subsample of HHs already visited on the previous days by their teams.

Estimating intervention coverage is a critical aspect of program monitoring and evaluation. While HH survey estimates are considered the gold standard, programmatic data can also be leveraged for this purpose. However, there is currently no guidance on optimal methods for collecting these programmatic data, and the choice of denominator (e.g., target population, visited individuals, or eligible individuals) significantly influences the reported coverage indicators, leading to variability in terminology and lack of comparability. In fact, a review of practices to estimate malaria intervention coverage found inconsistencies in the definition and reporting of coverage for different interventions [30]. Additionally, selecting an accurate and appropriate denominator remains a major challenge, particularly in the absence of a recently updated census, and methods for estimating denominators are often unclear or inconsistently documented across studies [30]. The use of the SI tool enabled us to obtain a HH denominator, with edits and additions made during fieldwork to ensure accuracy. To estimate the population denominator, we used the average household size obtained from the cross-sectional HH survey.

This approach was triangulated with administrative data (which applies a growth factor to the most recent census) to provide a range of denominator and coverage estimates, a potential approach recommended for estimating the denominator in settings where current and updated population data are not available [30]. Another option to refine denominator estimates is to adjust target population estimates for subsequent rounds using field-validated data from the first round [9]. 

The SI tool, with all structures marked on the map, greatly facilitated reaching all targeted HHs, helped the teams in the field locate and find each HH, and simplified the operationalization. The use of color-coding to indicate visit status facilitated knowing which households still needed to be visited, likely contributing to improved coverage. Real-time monitoring was also enhanced through the built-in dashboard. The use of similar platforms to enumerate structures and improve coverage of health interventions delivered at the community-level such as polio vaccination campaigns or IRS has been reported elsewhere [31–33], but enumerating HHs on the satellite images prior to field work can be costly and is very time consuming, so sustainability needs to be considered.

Timing of MDA is crucial, and WHO recommends implementation just before the malaria transmission season [9]. However, accurately identifying and initiating the campaign at the optimal moment can be challenging due to logistical delays and unusual weather patterns. For instance, even though the pMDA in Chidenguele was planned to start in November, just before the rainy season, it was eventually implemented when the high transmission season had already begun (December) due to logistical delays. Moreover, while the rainy season in southern Mozambique has historically occurred between November and March, a tendency for the rains to begin later in the year has been observed in recent years [34], further complicating the planning of intervention activities. In the 2022-2023 season, above-average rainfall occurred toward the later months, and tropical storm Freddy (which later intensified into a cyclone) made landfall twice between late February and mid-March, causing additional heavy rainfall [35]. These challenges highlight the need for flexibility in MDA planning to adjust the timing of the different rounds, taking into account changes in seasonality patterns and unexpected weather events.

Conducting multiple rounds is essential to maximize coverage, as shown by the HH survey results where 81% of the participants reported having participated in at least one of the rounds, especially given high population mobility and absenteeism. Some modeling analyses suggest that three rounds would result in better outcomes than two rounds because it maximizes the chances of finding individuals at least once [36]. Moreover, based on our experience, coverage in the first round is often suboptimal due to inexperience of the delivery teams, lower awareness of the population about the pMDA, or other factors, but it can serve as a pilot to both refine the planning and improve the denominators for the second and third rounds. In terms of the duration of each round, even though WHO recommends rounds to not last longer than one week [9], a huge number of resources and teams are needed to complete a round in this very short time, especially if some days for revisits and/or fixed points have to be included for mop-ups. Revisits and fixed points played a critical role in ensuring high coverage during the second round of pMDA in Chidenguele. This finding aligns with results from community-wide MDA trials for soil-transmitted helminths conducted in Benin, India, and Malawi [37], where adequate duration of MDA delivery was identified as a key factor in achieving high coverage.

MDA, like any other community-based intervention, requires intensive funding and human resources to be successfully implemented. Therefore, integration with other health interventions that also use a door-to-door approach could be explored to ensure resources are allocated in the most efficient way [38], For instance, the enumeration and mapping exercise conducted in the context of this pMDA campaign could be used by other interventions delivered at the HH-level to ensure that all houses in the area are reached, or MDA visits could be used to administer vitamin A supplementation or anthelmintic drugs. A systematic review of strategies to improve treatment coverage of community-based public health programs found that integration successfully increased coverage [39], with some examples including successful integration of LLIN distribution and MDA for lymphatic filariasis in Nigeria [40], or integration of measles and rubella vaccination with MDA for onchocerciasis and lymphatic filariasis in Tanzania [41]. In Senegal, a randomized controlled trial demonstrated that integrating MDA for helminth control and seasonal malaria chemoprevention was safe and feasible [42].

Overall, community participation in the pMDA campaign was good. Strong community and stakeholder engagement were essential, and the involvement of the local leaders at the community/neighborhood level, including the community guides, played a crucial role in terms of HHs welcoming and accepting the pMDA distribution teams, similar to what has been seen in other MDA studies [28]. 1.6% of individuals visited by pMDA teams refused to participate in pMDA1, and participation refusal increased to 8.1% in the second round. Even though we did not register reasons for refusal at the individual-level in the second round, results from the HH survey suggest that this increase could be due to knowing that someone suffered adverse events in the first round, which is also one of the main reasons for refusal at the HH-level [12]. Fear of adverse events was the second most common barrier to acceptability observed in pMDA1, in line with what has been observed in other MDA studies [28, 29]. 

In the context of a pMDA with programmatic coverages of 41% and 70% in the first and second rounds, respectively, we found weak evidence of a larger decrease in the post-intervention vs pre-intervention level change in the incidence of malaria immediately following the pMDA implementation period in the pMDA group versus the comparison group (IRR=0.66, p=0.074). However, no evidence was found of a difference in the incidence trend over time between the two groups (IRR=0.96, p=0.104). Findings from a systematic review assessing the impact of MDA for transmission reduction in very low to low transmission settings support these observations, indicating that MDA may have an immediate but short-term effect lasting 1-3 months post-intervention, but no effect at later times [43]. Moreover, although results from the HH survey showed that over 80% of the target population was treated in at least one of the two rounds, the time interval between rounds may have been too long to have an impact on transmission. Low coverage of LLIN use and IRS in the area (38% and 43% respectively, data from HH survey to be published elsewhere) may have further limited the impact. As recommended by the WHO, MDA should be implemented as part of a comprehensive package of interventions including high coverage of vector control, effective surveillance, and timely diagnosis and treatment [9].

This study has several limitations. First of all, potential issues related to data quality may have affected our results. It is possible that the programmatic data collected during the pMDA had data collection errors, inaccurate responses due to misunderstanding and/or reporting bias. Moreover, some of the results from the HH survey may have been influenced by social desirability or recall bias. Nevertheless, the coverage results obtained from both sources of data are fairly consistent, indicating an acceptable quality of the data collected during the campaign. Regarding the impact evaluation quasi-experimental design, even though a comparison group as similar as possible to the intervention group was selected, there might be selection bias due to the lack of randomization and the small number of HFs. Also, even though we adjusted for potential confounders in the cITS analysis, it is still possible that observed changes in incidence are influenced by residual confounding due to low quality data for the covariates or other confounders not considered in the analysis. In addition, because data on malaria cases and outpatient visits to the HF were obtained from DHIS2, it was not possible to ascertain whether reported cases were limited to the target populations within the intervention and comparison areas, as HFs may be used by individuals from neighboring catchment areas. This potential contamination may have influenced the estimated effect of the intervention. Moreover, there are also potential limitations related to the quality of the routine data collected through DHIS2, as these may not always be fully accurate or complete [44]. 

Due to concerns about data quality and potential reporting bias, routine surveillance data have not been widely used in impact evaluations. However, substantial investments have been made to strengthen HMIS, and the Global Technical Strategy for malaria 2016-2030 identifies surveillance as a core intervention, emphasizing the critical role of reliable, high-quality routine HMIS data [5, 45]. In fact, routine surveillance remains the primary source of information available to health managers and policymakers for evaluating programmatic interventions. Therefore, the use of these data in impact evaluations should be enhanced to build on the already available evidence on the impact of malaria MDAs using routine data [23, 30, 45–48]. This type of programmatic evaluations of interventions should be conducted routinely by national health programmes to assess the real impact of the interventions deployed within their strategic plans, should the quality and completeness of the available data allow it. Additional limitations of the study include lack of programmatic information on adherence to the second and third doses, and difficulties in selecting an accurate and appropriate population denominator for coverage estimates. 

## Conclusions

Although the implementation strategy and monitoring plan were successfully implemented, achieving the recommended 80% programmatic coverage for each round remains a significant challenge. This target was reached only when considering participation in either of the two rounds, highlighting the importance of conducting multiple rounds. In the second round, coverage was limited by unreached individuals, absences, ineligibility, and refusals. To improve uptake in future campaigns, it is essential to ensure all HHs are visited, plan for one or two additional days for revisits and/or fixed points, strengthen community engagement efforts, and potentially revisit exclusion criteria. Using a SI software greatly facilitated locating the targeted HHs and monitoring those that had to be revisited, although enumerating them prior to fieldwork was very time consuming, and sustainability should be considered. Overall, the pMDA campaign was accepted by the community, and community and stakeholder engagement proved essential to ensure a successful implementation. The daily monitoring of the implementation was also key, and strategies to mitigate challenges related to data quality should be put in place. Programmatic and HH survey data yielded similar coverage estimates. We found weak evidence of a difference in malaria incidence change immediately following the pMDA in the pMDA group versus the comparison group and no evidence of a difference in the incidence trend change over time between the two groups. However, limitations regarding the quality and availability of the routine data used might have influenced the results. The sustainability of implementing pMDA should be carefully considered by malaria programmes, particularly in light of its financial and human resource demands and its potential short-term impact. As such, pMDA may be best reserved for specific contexts, such as pre-elimination settings, emergency situations (though delivery models may differ), or when integrated with other community-based interventions. Moreover, it should be implemented together with high-coverage of core malaria interventions, such as case management, vector control and surveillance. Operational research studies and impact evaluations using routine data, such as the one presented here, can provide valuable evidence to countries on how to effectively scale-up strategies and inform evidence-based planning and decision-making. 

## Supplementary Information

Supplementary Material 1.

## Data Availability

The datasets supporting the conclusions of this article are available in the CORA Research Data Repository ( https://doi.org/10.34810/data2442 ). Access to these data is subject to approval by the Mozambican National Malaria Control Programme, the Manhiça Health Research Centre, and the Barcelona Institute for Global Health, and can be requested from the corresponding author upon reasonable request and with appropriate permissions.

## Abbreviations

AE: Adverse event
CHW: Community health worker
CISM: Centro de Investigação em Saúde de Manhiça (Manhiça Health Research Centre)
cITS: controlled interrupted time-series
DHAP: Dihydroartemisinin-piperaquine
DHIS2: District Health Information Software 2
DOT: Directly observed treatment
HF: Health facility
HFCA: Health facility catchment area
HH: Household
HMIS: Health management information system
IRR: Incidence risk ratio
IRS: Indoor residual spraying
ISGlobal: Barcelona Institute for Global Health
LLIN: Long-lasting insecticidal net
MDA: Mass drug administration
NMCP: National Malaria Control Program
pMDA: programmatic mass drug administration
pMDA1: programmatic mass drug administration round 1
pMDA2: programmatic mass drug administration round 2
SI: Satellite imagery
WHO: World Health Organization
