# Supplementary material for: Implementation, coverage and impact of a programmatic mass drug administration campaign for malaria in southern Mozambique
Source: BMC Public Health. 2026 Jul 3;26:2861. doi: 10.1186/s12889-026-28353-6 (PMC13647609; doi:10.1186/s12889-026-28353-6)
Supplement: Supplementary file 1 [file 12889_2026_28353_MOESM1_ESM.docx]

Implementation, coverage and impact of a programmatic mass drug administration campaign for malaria in southern Mozambique

# Supplementary Material

**S1 Fig A. pMDA1 coverage cascade at the household-level.** HH: household; SI: satellite imagery. Number of HHs from the census were estimated assuming 4 individuals per HH; number of HHs from SI were estimated from the number of HHs identified during the pre-implementation period on the satellite images and during the field work.

**S1 Fig B. pMDA2 coverage cascade at the household-level.** HH: household; SI: satellite imagery. Number of HHs from the census were estimated assuming 4 individuals per HH; number of HHs from SI were estimated from the number of HHs identified during the pre-implementation period on the satellite images and during the field work.

**S2 Fig. pMDA1 coverage cascade at the individual-level.** DOT: directly observed treatment; Indiv: individuals; MDA: mass drug administration; SI: satellite imagery. Expected population from census is based on the 2017 national census; expected population from SI was estimated assuming 4.08 individuals per HH.

**S3 Fig. Monthly incidence of passively-detected malaria cases by HFCA in intervention and comparison groups.** HFCA: health facility catchment area.

**S4 Fig A. Monthly number of outpatient visits to the HF in intervention and comparison groups.** HF: health facility. The number of outpatient visits to the HFs was obtained from the health management information system (DHIS2).

**S4 Fig B. Coverage of IRS by HFCA in intervention and comparison groups.** HFCA: health facility catchment area; IRS: indoor residual spraying. The IRS data were provided by district authorities.

**S4 Fig C. Mean rainfall by HFCA in intervention and comparison groups.** HFCA: health facility catchment area.

**S4 Fig D. Mean temperature by HFCA in intervention and comparison groups.** HFCA: health facility catchment area.

**S4 Fig E. EVI by HFCA in intervention and comparison groups.** EVI: enhanced vegetation index; HFCA: health facility catchment area.

**S1 Table. Definitions used for coverage outcomes.** Some of the definitions have been adapted from *Tanahashi, T. Health service coverage and its evaluation. Bull World Health Organ 56, 295–303 (1978).*

| **Outcome** | **Programmatic data (numerator/denominator)** | **Cross-sectional household survey data** |
| --- | --- | --- |
| **Household-level** | | |
| **Availability coverage** | Number of HHs that were reached^a^/number of target HHs | Number of individuals that reported that their HH was reached/number of individuals surveyed |
| **Accessibility coverage** | Number of HHs visited^b^/number of target HHs | - |
| **Empty households** | Number of HH that were empty/number of HH that were reached | - |
| **Acceptability coverage** | Number of HHs that accepted to participate in the campaign/number of HHs visited | - |
| **Participation refusal** | Number of HHs that refused to participate in the campaign/number of HHs visited | - |
| **Individual-level** | | |
| **Availability coverage** | Number of individuals reached^c^/target population | Number of individuals that reported being reached during pMDA/number of individuals surveyed |
| **Accessibility coverage** | Number of individuals visited^d^/target population | Number of individuals that reported being visited/number of individuals surveyed |
| **Programmatic or contact coverage^e^** | Number of individuals that received DHAP/target population | Number of individuals that reported having received DHAP/number of individuals surveyed |
| **Effective coverage** | Number of individuals that received DHAP/number of individuals reached | Number of individuals that reported having received DHAP/number of individuals surveyed reporting being reached |
| **Operational coverage** | Number of individuals that received DHAP/number of individuals visited | Number of individuals that reported having received DHAP/number of individuals surveyed reporting being visited |
| **Treatment coverage among individuals that accept** | Number of individuals that received DHAP/number of individuals that accepted to participate | Number of individuals that reported having received DHAP/number of individuals surveyed that reported having accepted to participate |
| **Treatment coverage among eligible individuals** | Number of individuals that received DHAP/number of eligible individuals | - |
| **Absences** | Number of absent individuals/number of individuals reached | Number of individuals that reported being absent/number of individuals surveyed reporting being reached |
| **Ineligibility among individuals that accept** | Number of ineligible individuals/number of individuals that accepted to participate | - |
| **Acceptability coverage** | Number of individuals that accepted to participate/number of individuals visited | Number of individuals that reported having accepted to participate/number of individuals surveyed that reported being visited |
| **Participation refusal** | Number of individuals that refused to participate/number of individuals visited | Number of individuals that reported having refused to participate/number of individuals surveyed that reported being visited |

^a^ Includes HHs with someone present as well as empty houses at the moment of the visit

^b^HHs where the pMDA team was able to talk to someone, including HHs that accepted to participate in the campaign and HHs that refused

^c^Includes individuals that accepted to participate in the campaign, individuals that refused, and absent individuals

^d^Includes present individuals that accepted or that refused

^e^Also termed “distribution coverage”

**S1 Table. Distribution of the population treated by age and sex in pMDA1 and by age in pMDA2.** As data collection in pMDA2 was aggregated at the household-level, no data are available by sex, and age groups are available according to the drug presentations.

|  | **pMDA1** | | | **pMDA2** |
| --- | --- | --- | --- | --- |
| **Age (years)** | **Female**  **(% of total)** | **Male**  **(% of total)** | **Total**  **(% of total)** | **Total**  **(% of total)** |
| **6 months - <2** | 428  (1.8) | 447  (1.8) | 875  (3.6) | 1,541  (3.7) |
| **2-4** | 1,067  (4.4) | 1,033  (4.2) | 2,100  (8.6) | 3,212  (7.8) |
| **5-7** | 1,139  (4.7) | 1,139  (4.7) | 2,278  (9.4) | 3,860  (9.3) |
| **8-13** | 2,710  (11.1) | 2,769  (11.3) | 5,479  (22.4) | 8,839  (21.4) |
| **14-19** | 1,920  (7.9) | 1,866  (7.6) | 3,786  (15.5) | 23,868  (57.8) |
| **20-29** | 1,557  (6.4) | 894  (3.7) | 2,451  (10.1) |  |
| **30-39** | 1,282  (5.2) | 571  (2.3) | 1,853  (7.5) |  |
| **40-49** | 1,152  (4.7) | 515  (2.1) | 1,667  (6.8) |  |
| **50-59** | 956  (3.9) | 447  (1.8) | 1,403  (5.7) |  |
| **≥60** | 1,155  (4.7) | 727  (3.0) | 1,882  (7.7) |  |
| **Unknown** | 488  (2.0) | 171  (0.7) | 663*  (2.7) | 0  (0.0) |
| **All ages** | **13,854**  **(56.8)** | **10,579**  **(43.2)** | **24,437***  **(100.0)** | **41,320**  **(100.0)** |

NI: no information; pMDA: programmatic mass drug administration

*Includes 4 individuals for whom age and sex in pMDA1 are unknown.

**S1 Appendix.** **Questionnaire used to collect individual-level data during pMDA1 translated into English.** Original version in Portuguese available upon request. pMDA: programmatic mass drug administration.

| **DOOR-TO-DOOR HOUSEHOLD SURVEY** | | |
| --- | --- | --- |
| **MODULE 1. Information of the inquirer** | | |
| 1.1 | Supervisor code | *[Select in the dropdown list]* |
| 1.2 | Register code | *[Select in the dropdown list]* |
| 1.3 | Distributor code | *[Select no dropdown list]* |
| 1.4 | Name of the Health Facility | *[Select no dropdown list]* |
| 1.5 | Round number | □ Round 1 □ Round 2 |
| **MODULE 2. Identification of the household, household members and consent** | | |
| 2.1 | Household ID | \|__\|__\|__\|__\|__\|__\|__\|__\|__\| |
| 2.2 | *Record your GPS coordinates. Stand in front of the household door while you record the GPS data.* | |
| 2.3 | Full name of head of household (or another household representative)  *If the participant/member does not want to give their name, write NA.* | \|__\|__\|__\|__\|__\|__\|__\|__\|__\| |
| 2.4 | Is this your first or second visit to this household? | □ 1^st^ visit □ 2^nd^ visit |
| 2.5 | If “2^nd^ visit”, Why did you have to come back? | □ Refusal □ Absence |
| 2.6 | Was the visit made (were you able to speak to the head of household or representative)? | □ Yes □ No |
| 2.7 | If "No", specify the reason: | □ Absence of the head of household/representative  □ Household empty or with no one at the moment of the visit  □ Other: \|__\|__\|__\|__\|__\|__\| |
| 2.8 | *If 2.6 "No": Go to Module 3. End of visit (refused).* | |
| 2.9 | Total number of PRESENT or ABSENT people living permanently in the household (sleeping at least 3 nights a week in the household this month).  *If the participant/member does not want to answer, enter 99.* | \|__\|__\| |
| 2.10 | Number of visitors or temporary guests who are in the household now.  *If the participant/member does not want to answer, enter 99.* | \|__\|__\| |
| 2.11 | DOES THE HOUSEHOLD HEAD (OR ANOTHER HOUSEHOLD REPRESENTATIVE) ACCEPT THE PARTICIPATION OF THE HOUSEHOLD? | □ Yes □ No, household refusal |
| 2.12 | If “No”, Reason for refusal: | □ Someone in the household suffered adverse events in the previous round  □ Doesn't see the need or benefit in taking the medicine  □ Fear of side effects  □ Treatment is too long or complicated  □ Interferes with routine habits (e.g. alcohol consumption)  □ Distrust of government or organizations  □ Refusal to obey leadership  □ Local political tension  □ Doesn't want to answer  □ Other: \|__\|__\|__\|__\|__\|__\| |
| 2.13 | If "Round 2": Did the household receive a visit from the malaria MDA team during Round 1 (December)? | □ Yes □ No □ Don’t know  □ Don’t want to answer |
| 2.14 | - *If 2.11 "No, household refused": Go to Module 3.* - *If 2.11 "Yes": Save the data, close the Household Survey and open the Participant/Member Survey.* | |
| **MODULE 3. End of the visit (visit not done or refused)** | | |
| 3.1 | *If 2.6 "No, visit not made" or if 2.11 "No, household refused": Fill in the household data on the Daily Control Sheet of medicines distributed by field workers (Annex 2a), in the section "Total empty/no one at time of visit/refused households".* | |
| 3.2 | *If 2.6 "No”: Place the yellow sticker on the household door (Annex 8).* | |
| 3.3 | *If 2.11 "No, household refused": Place the corresponding sticker on the household door (Annex 8).* | |
| 3.4 | *If 2.6 "No” or if 2.11 "No, household refused": Fill the status of the household in the Reveal app.* | |

| **DOOR-TO-DOOR PARTICIPANT/MEMBER SURVEY** | | |
| --- | --- | --- |
| **MODULE 1. Information of the inquirer** | | |
| 1.1 | Supervisor code | *[Select in the dropdown list]* |
| 1.2 | Register code | *[Select in the dropdown list]* |
| 1.3 | Distributor code | *[Select no dropdown list]* |
| 1.4 | Name of the Health Facility | *[Select no dropdown list]* |
| 1.5 | Round number | □ Round 1 □ Round 2 |
| 1.6 | Type of visit | □ Door-to-door  □ Distribution point |
| 1.7 | If “Distribution point”: | □ Market  □ Church  □ School  □ Health Facility  □ Institution  □ Other: \|__\|__\|__\|__\|__\|__\| |
| **Only if 1.6 “door-to-door”**  **MODULE 2. Identification of the household** | | |
| 2.1 | Household ID | \|__\|__\|__\|__\|__\|__\|__\|__\|__\| |
| 2.2 | *Record your GPS coordinates. Stand in front of the household door while you record the GPS data.* | |
| 2.3 | Name of the household head | \|__\|__\|__\|__\|__\|__\|__\|__\|__\| |
| **MODULE 3. Identification of the participant and consent** | | |
|  | *Only if 1.6 "door-to-door":*  *Ask these questions to each and every member residing in the household or visiting, PRESENT or ABSENT at the time of the visit.* | |
| 3.1 | Name of the participant/member  *If the participant/member does not want to give their name, write NA.* | \|__\|__\|__\|__\|__\|__\|__\|__\|__\| |
| 3.2 | *Only if 1.6 "door-to-door":*  Does the participant/member live permanently in the household? (sleeps at least 3 nights a week in the household this month) | □ Yes, permanent resident  □ No, visitor or non-permanent resident |
| 3.3 | Age (Years) | \|__\|__\| |
| 3.4 | If age 0, Age (Months) | \|__\|__\| |
| 3.5 | Gender  *If the participant/member doesn't want to answer, check the NA box.* | □ Female  □ Male  □ NA |
| 3.6 | Is this your first or second visit to this member? | □ 1^st^ visit □ 2^nd^ visit |
| 3.7 | If “2^nd^ visit”, Why did you have to come back? | □ Refusal □ Absence |
| 3.8 | *Only if 1.6 "door-to-door":*  The participant/member is present in the household at the moment of the visit? | □ Yes □ No, absent |
|  | *If "No, absent": End of the individual's participation. Continue with Module 6.* | |
| 3.9 | THE PARTICIPANT/MEMBER ACCEPTS TO PARTICIPATE? | □ Yes □ No, refuses |
| 3.10 | If “No”, Reason for refusal: | □ Someone known suffered adverse events in the previous round  □ Doesn't see the need or benefit in taking the medicine  □ Fear of side effects  □ Treatment is too long or complicated  □ Interferes with routine habits (e.g. alcohol consumption)  □ Distrust of government or organizations  □ Refusal to obey leadership  □ Local political tension  □ Doesn't want to answer  □ Other: __\|__\|__\|__\|__\|__\| |
|  | - *If 3.9 "No, refusal" and 1.6 "door-to-door": End of the individual's participation. Continue with module 6.* - *If 3.9 "No, refusal" and 1.6 "fixed distribution point": End of individual's participation. Continue with module 7.* | |
| 3.11 | If "Round 2": Did the participant/member take the medication during round 1 (November/December) of the MDA campaign? | □ Yes □ No  □ Doesn’t know |
| 3.12 | If "Yes": For how many days did the participant take the medication? | □ Only took the first dose in front of the inquirer  □ Two (2) days in total  □ Three (3) days in total  □ Doesn’t know  □ Doesn’t remember |
| 3.13 | If "Yes": Did the participant experience any adverse effects/reactions during or after taking the medication? | □ No  □ Doesn’t know  □ Doesn’t remember  □ Allergic reaction  □ Cardiac problem  □ Dizziness  □ Vomiting  □ Headache  □ Arthralgia  □ Malaise  □ Diarrhoea  □ Other: \|__\|__\|__\|__\|__\|__\| |
| 3.14 | If "Yes": How many days after taking the medication did the adverse effect start? | □ On the same day  □ 1 day after  □ 2 days after  □ 3 or more days after  □ Doesn’t know  □ Doesn’t remember |
| **MODULE 4. Inclusion and exclusion criteria for the MDA** | | |
|  | *If necessary, use the flowchart for applying the exclusion criteria (Appendix 5) to assess the participant's eligibility.* | |
| 4.1 | Is the participant/member a baby under 6 months old?   - *If "Yes", proceed to question 5.1* | □ Yes □ No |
| 4.2 | Is the participant/member currently seriously ill (bedridden or have an illness that prevents from carrying out day-to-day activities (such as cooking, etc.))?   - *If "Yes", proceed to question 5.1* | □ Yes, seriously ill □ No |
| 4.3 | Has the participant/member received DHAP in his/her household or in another household, fixed point or school (if children) during the last 30 days?   - *If "Yes", proceed to question 5.1* | □ Yes, medication received in the household, at a fixed distribution point or at school  □ No |
| 4.4 | Has the participant/member had a fever (hot body) in the last 24 hours?   - *If "Yes", proceed to question 5.1* | □ Yes, fever in the last 24 hours  □ No |
| 4.5 | Has the participant/member taken antimalarials in the last 15 days?   - *If "Yes", proceed to question 5.1* | □ Yes, antimalarial treatment in the last 15 days  □ No |
| 4.6 | Is the participant/member currently receiving intermittent preventive treatment (IPT) for malaria in pregnancy with sulfadoxine-pyrimethamine (SP)?   - *If "Yes", proceed to question 5.1* | □ Yes □ No |
| 4.7 | Is the participant/member currently taking any other medication?   - *If "Yes", ask them to show it to you.* | □ Yes □ No |
| 4.8 | If "Yes", is the participant taking one of these contraindicated drugs??   - *If "Yes", proceed to question 5.1* | □ Yes, cotrimoxazole  □ Yes, erythromycin  □ Yes, azithromycin  □ Yes, clarithromycin  □ Yes, lumefantrine (Coartem)  □ Yes, quinine  □ Yes, toconazole  □ Yes, fluconazole  □ Yes, mefloquine  □ Yes, haloperidol  □ Yes, promethazine  □ Yes, chlorpromazine  □ No |
| 4.9 | Does the participant/member have a known heart condition?   - *If "Yes", proceed to question 5.1* | □ Yes, cardiac problem  □ No |
| 4.10 | Has the participant/member ever had an allergic reaction after taking antimalarial drugs including artemisinin or piperaquine, or does he/she have a known allergy to these drugs?   - *If "Yes", proceed to question 5.1* | □ Yes, allergy  □ No |
| 4.11 | *If 3.3 "12-49 years" and 3.4 "Female":*  Are you pregnant?  *Ask gently, in your own words and in a private setting.* | □ Yes, reports being pregnant  □ No, does not report being pregnant  □ Doesn’t know  □ Prefers not to answer |
| 4.12 | - *If 4.11 "No, does not report being pregnant": Explain to the woman that these antimalarial drugs should not be taken during the first trimester of pregnancy and that it is important to say if she thinks she might be pregnant. If the woman thinks she might be pregnant, change the answer to the previous question.* - *If 4.11 "No, does not report being pregnant", continue with question 5.3.* - *If 4.11 "Doesn't know" or "Prefers not to answer", continue with question 5.1.* | |
| 4.13 | If 4.11 "Yes": How long has she been pregnant? | □ 3 months or less (from 1 to 12 weeks, probably not visibly pregnant)  □ More than 3 months (from week 13, probably visibly pregnant)  □ Doesn’t know/Unknown |
| **MODULE 5. Administration of the medication** | | |
| 5.1 | - *If 4.1-4.11 "Yes"; or if 4.11 is "Doesn’t know" or "Prefers not to answer": DO NOT administer the medicine. End of participation.* - *If 4.2 or 4.4 "Yes": DO NOT administer the medicine. Fill in the reasons for referral on the Referral Guide to the Health Facility (Appendix 7) and refer the participant to the Health Facility.* - *If 1.6 "door-to-door", continue with Module 6.* - *If 1.6 "fixed distribution point", continue with Module 7.* | |
| 5.2 | *Explain how to take the medicine. If necessary, use section 7 in the Procedures for Mass Drug Administration (Annex 1a) as a support.* | |
| 5.3 | Does the participant/member accept to take the medicine? | □ Yes □ No |
| 5.4 | If "No", Why not? | □ Suffered adverse events in the previous round  □ Does not see the need to take the medicine  □ Does not want to take a medicine he/she does not know  □ Does not believe the medicine prevents malaria  □ Treatment is too long or complex  □ Religious or cultural beliefs  □ Interferes with routine habits (e.g. alcohol consumption)  □ Fear of the medication or side effects  □ Heard negative rumours about the medication  □ Does not trust the study team  □ Does not know the study team  □ Does not understand what the medication is for  □ Other: \|__\|__\|__\|__\|__\|__\| |
|  | - *If 5.3 "No", End of participation.* - *If 1.6 "door-to-door", continue with Module 6.* - *If 1.6 "fixed distribution point", continue with Module 7.* | |
| 5.5 | *If 5.3 "Yes":*   - *If age 6 - ≤11 months (5-7 kg): Give the participant 1 box of 3 tablets 20mg/160mg (Pink). Crush 1 tablet, dissolve in water and observe the taking of the first dose (1 tablet).* - *If age is 1 year (8-10 kg): Give the participant 2 boxes of 3 tablets 20mg/160mg (Pink). Crush one and a half tablets, dissolve water and observe the taking of the first dose (1.5 tablets).* - *If age ≥2 - 4 years (11-16 kg): Give the participant 1 pack of 3 tablets 40mg/320mg (Orange). Crush one tablet, dissolve in water and observe the taking of the first dose (1 tablet).* - *If age ≥ 5 - 7 years (17-24 kg): Give the participant 1 Box of 6 tablets 40mg/320mg (Orange). Crush one and a half tablets, dissolve in water and observe the taking of the first dose (1.5 tablets).* - *If age ≥ 8 - 13 years (25-36 kg): Give the participant 1 box of 6 tablets 40mg/320mg (Orange). Observe the taking of the first dose (2 tablets).* - *If age ≥14 years (37+ kg): Give the participant 1 Box of 9 tablets 40mg/320mg (Blue). Observe the taking of the first dose (3 tablets).* | |
| 5.6 | What dosage of medication did you give? | □ One box of 3 tablets 20 mg/160 mg  □ Two boxes of 3 tablets 20 mg/160 mg  □ A carton of 3 tablets 40 mg/320 mg  □ A box of 6 tablets 40 mg/320 mg  □ A box of 9 tablets 40 mg/320 mg |
| 5.7 | Did the participant/member take the first dose in the presence of the distributor? | □ Yes □ No |
| 5.8 | If "No", why not? | □ There was no water available  □ The participant couldn't swallow  □ Other: \|__\|__\|__\|__\|__\|__\| |
|  | *After receiving treatment with DHAP, observe the participant/member for a minimum of 10 minutes (up to 30 minutes if time allows).* | |
| **Only if 1.6 “door-to-door”**  **MODULE 6. End of the visit (participant)** | | |
| 6.1 | Have you collected the data of all the members/participants living in the household or visiting (present and absent)? | □ Yes □ No |
| 6.2 | - *If "No", Save the data, add a new file to the Member Survey folder and start the questionnaire for the next participant/member.* - *If "Yes", continue with Module 7.* | |
| **MODULE 7. End of visit (completed household)/End of administration** | | |
| 7.1 | *Fill in the MDA data on the Daily Control Sheet for medicines distributed by field workers (Annex 2a).* | |
| 7.2 | *If "Round 1" and if 1.6 "door-to-door": Give the Medication Card (Annex 4) to the head of the household. Write on the card the household ID, the date and time of administration of doses 1, 2 and 3 (round 1) and also the expected date for dose 1 of round 2.* | |
| 7.3 | *If "Round 1" and if 1.6 "fixed point": Give the Medication Card (Annex 4) to the participant/member. Write on the card the name of the participant/member, the date and time of administration of doses 1, 2 and 3 (round 1) and also the expected date for dose 1 of round 2.* | |
| 7.4 | If 1.6 "fixed distribution point": Ask the participant/member to come back if they vomit in the next 30 minutes. If they vomit, repeat the treatment. | |
| 7.5 | If 1.6 "door-to-door": Has the participant/household member vomited? | □ Yes □ No |
| 7.6 | If “Yes”, Did you repeat the treatment? | □ Yes □ No |
| 7.7 | If 1.6 "door-to-door": After obtaining permission from the household head, place the corresponding sticker on the household door (Appendix 8). | |
| 7.8 | If 1.6 "door-to-door": Write the date on the sticker and, if you place the yellow sticker, write how many participants/members of the household were absent or refused during the visit. | |
| 7.9 | If 1.6 "door-to-door": Mark the status of the household in the Reveal app. | |

**S2 Appendix. Questionnaire used to collect household-level aggregated data during pMDA2 translated into English.** Original version in Portuguese available upon request. pMDA: programmatic mass drug administration.

| **DOOR-TO-DOOR SURVEY** | | |
| --- | --- | --- |
| **MODULE 1. Information of the inquirer** | | |
| 1.1 | Supervisor code | *[Select in the dropdown list]* |
| 1.2 | Team code | *[Select in the dropdown list]* |
| 1.3 | Round number | □ Round 1 □ Round 2 |
| **MODULE 2. Identification of the household, household members and consent** | | |
| 2.1 | Household ID | \|__\|__\|__\|__\|__\|__\|__\|__\|__\| |
| 2.2 | *Record your GPS coordinates. Stand in front of the household door while you record the GPS data.* | |
| 2.3 | Full name of household head (or another household representative)  *If the participant/member does not want to give their name or the household is empty, write NA.* | \|__\|__\|__\|__\|__\|__\|__\|__\|__\| |
| 2.4 | Was the visit made (were you able to speak to the head of household or representative)? | □ Yes □ No |
| 2.5 | If “No”, Specify the reason: | □ Absence of the head of household/representative  □ Empty household or no one at the moment of the visit  □ Other: \|__\|__\|__\|__\|__\|__\| |
| 2.6 | Is this your first attempt to visit this household this round, or is it a recovery visit? | □ 1^st^ visit □ 2^nd^ visit (recovery visit) |
| 2.7 | If “2^nd^ visit”, Why did you have to come back? | □ Refusal by some members (partial refusal)  □ Absence of some members (partial absence)  □ Refusal of the whole household  □ Complete household absent (empty household) |
| 2.8 | If "Partial refusal or Partial absence": How many people who were not treated at the 1st visit are now present and agree to participate? | \|__\|__\| |
|  | *If 2.4 is "No": End of survey* | |
|  | *Use the Household registration table (Appendix 14a) to answer the following questions:* | |
| 2.9 | TOTAL number of PERMANENT RESIDENTS (present and absent) and VISITORS present in this household  *If the participant/member does not want to answer, enter 99.* | \|__\|__\| |
| 2.10 | Total number of people in this household who are currently ABSENT  *If the participant/member does not want to answer, enter 99.* | \|__\|__\| |
| 2.11 | Total number of people who received the treatment in Round 1  *If the participant/member does not want to answer, enter 99.* | \|__\|__\| |
| 2.12 | DOES THE HOUSEHOLD HEAD (OR ANOTHER HOUSEHOLD REPRESENTATIVE) ACCEPT THE HOUSEHOLD'S PARTICIPATION? | □ Yes □ No, refusal of the whole household |
| 2.13 | If “No”, Reason for refusal: | □ Someone in the household suffered adverse events in the previous round  □ Does not see the need or benefit of taking the medicine  □ Fear of side effects  □ Treatment is too long or complicated  □ Interferes with routine habits (e.g. alcohol consumption)  □ Does not want to take it on an empty stomach  □ Distrust of government or organizations  □ Refusal to obey leadership  □ Local political tension  □ Does not want to answer  □ Outro: \|__\|__\|__\|__\|__\|__\| |
|  | *If 2.12 is "No": End of survey* | |
| 2.14 | Total number of people treated aged 6 - 11 months | \|__\|__\| |
| 2.15 | Total number of people treated aged 12 - 23 months | \|__\|__\| |
| 2.16 | Total number of people treated aged 2 - 4 years | \|__\|__\| |
| 2.17 | Total number of people treated aged 5 - 7 years | \|__\|__\| |
| 2.18 | Total number of people treated aged 8 - 13 years | \|__\|__\| |
| 2.19 | Total number of people treated over 14 years old | \|__\|__\| |
| 2.10 | Total number of people who have been EXCLUDED (because they are not eligible) | \|__\|__\| |
| 2.21 | Total number of people who REFUSED | \|__\|__\| |

| **DISTRIBUTION POINT SURVEY** | | |
| --- | --- | --- |
| **MODULE 1. Information of the inquirer** | | |
| 1.1 | Supervisor code | *[Select in the dropdown list]* |
| 1.2 | Team code | *[Select in the dropdown list]* |
| 1.3 | Round number | □ Round 1 □ Round 2 |
| **MODULE 2. Summary distribution point (end of the day)** | | |
|  | *Use the Fixed point recording table (Appendix 14b) to answer the following questions:* | |
| 2.1 | TOTAL number of people present at the fixed point | \|__\|__\|__\|__\| |
| 2.2 | Total number of people whose households were visited in this round | \|__\|__\|__\|__\| |
| 2.3 | Total number of people treated aged 6 - 11 months | \|__\|__\| |
| 2.4 | Total number of people treated aged 12 - 23 months | \|__\|__\| |
| 2.5 | Total number of people treated aged 2 - 4 years | \|__\|__\| |
| 2.6 | Total number of people treated aged 5 - 7 years | \|__\|__\| |
| 2.7 | Total number of people treated aged 8 - 13 years | \|__\|__\| |
| 2.8 | Total number of people treated over 14 years old | \|__\|__\| |
| 2.9 | Total number of people who have been EXCLUDED (because they are not eligible) | \|__\|__\| |
| 2.10 | Total number of people who REFUSED | \|__\|__\| |
| 2.11 | Total number of people who received the treatment in Round 1 | \|__\|__\| |

**S3 Appendix. Operationalization and implementation strategy of the pMDA.** pMDA: programmatic mass drug administration.

Human resources:

106 fieldworkers (53 teams) participated in pMDA1. In an effort to increase coverage in pMDA2, the number of fieldworkers was increased to 144 (72 teams). Each team consisted of one register (responsible for recording the information about the administration) and one distributor (responsible for providing the information about the pMDA campaign and administering the medication). Given that each team was accompanied by a community guide, there were 53 community guides in pMDA1 and 72 in pMDA2. The number of community mobilizers was also increased from the first round to the second, from 17 to 46, so as to have one community mobilizer in each neighbourhood. Each supervisor (8 in pMDA1 and 9 in pMDA2) was responsible for a maximum of 8 teams. There were 28 coordinators, including representatives of the National Malaria Control Program, the Medical Chiefs and Malaria Focal Points at the province- and district-level, the district Health Director, the district Pharmacist, the district Pharmacovigilance Focal Point, and the district Community Health Focal Point.

The coordinators and supervisors were trained before the start of pMDA1 following the Implementation Manual covering all aspects of the pMDA campaign and tablets with the applications to be used by the teams in the field.

Fieldworkers were trained prior to each pMDA round by coordinators and supervisors. The training combined theoretical and practical sessions (i.e., role-play and simulations) that covered all the procedures to be performed in the field. A post-test was conducted to inform the selection of the supervisors and the fieldworkers.

Community mobilizers also received a specific training, which placed special emphasis on the key messages around the pMDA and the importance of taking the medication, as well as on how to avoid and react to refusals and rumours.

Implementation strategy:

The campaign lasted for 12 days in the first round and 15 days in the second round. Initially, a “scanning” strategy was used, which consisted in allocating all teams of fieldworkers in all the areas (i.e., villages) to be visited each day in an effort to achieve the maximum coverage in one day in each village and then moving on to the neighbouring village. However, during the first days of pMDA1, a high number of households were not reached due to lack of time and many individual absences were registered in the visited households, so the delivery strategy was switched to a “decentralized” strategy. This strategy consisted in allocating a team of fieldworkers in a specific neighbourhood (or smaller area depending on the population density) during the entire pMDA campaign, thus facilitating recovery visits for absent individuals and households, and therefore having teams across the whole implementation area working in parallel throughout the campaign.

The main pMDA delivery mode was door-to-door visits, with a household target per team per day of 25 in pMDA1 and 15 in pMDA2. This target was decreased for pMDA2 after realizing that fieldworkers were not able to reach their target due to long distances between households and time spent in each visit. Additionally, one and two days of fixed points were implemented in pMDA1 and pMDA2, respectively, as a mop-up strategy. These fixed points were placed in markets, churches, health facilities, schools, neighbourhood committees, etc.

Every day, all coordinators, supervisors and fieldworkers met at the main health facility, both in the morning and at the end of the day, to pick up and return the materials and the medicines, plan the work of the day, and discuss any issues encountered during field work.

Mapping of the households and navigation in the field:

The Reveal® platform (Akros, <https://revealprecision.com/>) was used to support the implementation of the pMDA campaign in the field. Prior to implementation, all structures (i.e., households, buildings) in the administrative post of Chidenguele were enumerated and mapped using satellite imagery (SI). The map was divided into areas of similar size and transferred into the Reveal® application, which was installed on the fieldworkers’ tablets. Each fieldworker was provided an agenda with the areas (with its mapped structures) to visit every day. Reveal® also had Global Positioning System navigation to help locate every targeted household once in the field. When the fieldworkers reached a mapped structure, they were able to validate whether it was an eligible household for the pMDA or not (i.e., if the structure was not a household or was an abandoned structure). They were also able to add new structures that did not appear on the map, thus providing an accurate household target denominator. Fieldworkers marked the structures on the map as ineligible (black), HH where the pMDA visit was completed (green), HH where the visit was partially completed (orange), or HH that refused to participate or with long-term absences (red) (**Figure A**).

**Figure A.** Reveal® map

Procedures in the household:

Once in the household, delivery teams introduced themselves, explained the objectives of the pMDA campaign and provided information about the procedures to be conducted in the household. After obtaining oral consent to conduct the visit, eligibility criteria were verified for all household members and visitors. Fieldworkers were specifically instructed to ask women of reproductive age about whether they could be pregnant, gently and in a private setting. No pregnancy tests were performed. Women unsure about their pregnancy status and those who did not want to answer were excluded. All participants eligible and consenting to participate received the first dose of the antimalarial medicine according to their age under direct observation. For children under five years of age, the tablets were crushed and dissolved in water. After having taken the medicine, participants were observed for at least 10 minutes to monitor that the medication was well tolerated. In case of vomiting, a second dose was administered. Counselling about how to take the second and third doses was provided, including the need and importance of completing the full treatment. Participants were also informed about the most common side effects associated with the antimalarial medicine and were asked to report and seek care at the nearest health facility in case of any moderate or severe adverse event. The need to continue using other malaria prevention control tools such as bednets was also emphasized by the delivery teams. A medication card was provided to participating households (and individuals presenting to the fixed delivery points) as proof that they had received the first dose of the treatment. It also contained information about which dosage corresponded to each household member, the date and time that the first dose was administered, and the date and time that the second and third dosages should be taken. The most common adverse events associated with the medication were listed on the card, as well as what to do in case of any participant experiencing any adverse event after taking the medication. A sticker was placed on the door of the household to indicate that a visit had been done and the status of the pMDA (green for pMDA completed, yellow for pMDA partially completed – meaning that there were some household members that were absent at the moment of the visit and that delivery teams needed to go back –, and red for households that refused to participate in the campaign or that were absent for a long period of time). Visitation status was also marked on the Reveal® map.

A standardized electronic questionnaire using DHIS2 was filled out at each household (**S1 Appendix** and **S2 Appendix**), regardless of whether the visit could be completed or not.

Progress monitoring:

All the data collected in the field was visualized through dashboards to support progress monitoring. Reveal® dashboards were used to allocate the delivery teams, monitor which structures were already visited and plan revisits. DHIS2 dashboards were also reviewed daily to monitor coverage by area, supervisor and team and to identify areas with low coverage or high rates of absences or refusals. Coordinators reviewed the dashboards every evening and produced a report summarizing team performance, progress towards coverage targets and possible data-related errors, which was discussed in a meeting between coordinators and supervisors every morning before going to the field.

Stock control:

In an effort to facilitate drug stock control, the pharmacist registered the number of packages delivered to each supervisor every morning using an electronic questionnaire. Each supervisor also registered the number of packages received and the number of packages delivered to each team. At the end of the day, each delivery team calculated how many people they treated and how many packages they should have administered and compared the result to the number of remaining packages they had. In case of any mismatch, it was discussed and reviewed with the supervisor. Supervisors returned the leftover packages to the pharmacist, and both registered the total packages returned and packages received, respectively, on an electronic questionnaire. These data also populated dashboards to visualize medicine stock management and detect any issues.

**S4 Appendix.** **cITS model structure and description.** *Reference: Linden A. Conducting interrupted time-series analysis for single- and multiple-group comparisons. The Stata Journal. 2015;15:480–500.*

The regression model was as follows:

*Y_t_ =* β*_0_ +* β*_1_T_t_ +* β*_2_X_t_ +* β*_3t_X_t_T_t_ +* β*_4_Z +* β*_5_ZT_t_ +* β*_6_ZX_t_ +* β*_7_ZX_t_T_t_ + ∑Covariates +ɛ_t_*

Y_t_ corresponds to the malaria cases at the HFCA-level by month (t). β_0_ to β_3_ represent the comparison group and β_4_ to β_7_ represent the pMDA group (Z). β_0_ represents the incidence in the comparison group at the start of the study, β_1_ the incidence time trend in the comparison group prior to the intervention, T_t_ the number of months since the beginning of the study period, β_2_ the change in the incidence level in the comparison group immediately after pMDA, X_t_ the pre- or post-intervention period (0 for pre- and 1 for post-intervention), β_3_ the change in the incidence trend between the post and the pre-intervention periods in the comparison group, β_4_ and β_5_ the difference in the incidence level (intercept) and the incidence time trend (slope) between the two groups before the intervention, respectively, β_6_ the change in incidence between post- and pre-intervention periods in the pMDA vs the comparison group, immediately following the introduction of the pMDA (level change), and β_7_ the change in the incidence trend (slope) between post and pre-intervention periods in the pMDA vs the comparison group (trend change)*. ɛ*_t_ corresponds to the error term.

The table below represents an example for two generic health facility catchment areas (one in the intervention group and one in the comparison group) with two observations pre-intervention and four observations post-intervention. The dotted rows indicate the pMDA in month 3. *Reference: Linden A. Conducting interrupted time-series analysis for single- and multiple-group comparisons. The Stata Journal. 2015;15:480–500.*

**Data structure:**

| **ID** | ***T*** | ***X*** | ***XT*** | ***Z*** | ***ZT*** | ***ZX*** | ***ZXT*** |
| --- | --- | --- | --- | --- | --- | --- | --- |
| **I** | 1 | 0 | 0 | 1 | 1 | 0 | 0 |
| **I** | 2 | 0 | 0 | 1 | 2 | 0 | 0 |
| **I** | 3 | 1 | 0 | 1 | 3 | 1 | 0 |
| **I** | 4 | 1 | 1 | 1 | 4 | 1 | 1 |
| **I** | 5 | 1 | 2 | 1 | 5 | 1 | 2 |
| **I** | 6 | 1 | 3 | 1 | 6 | 1 | 3 |
| **C** | 1 | 0 | 0 | 0 | 0 | 0 | 0 |
| **C** | 2 | 0 | 0 | 0 | 0 | 0 | 0 |
| **C** | 3 | 1 | 0 | 0 | 0 | 0 | 0 |
| **C** | 4 | 1 | 1 | 0 | 0 | 0 | 0 |
| **C** | 5 | 1 | 2 | 0 | 0 | 0 | 0 |
| **C** | 6 | 1 | 3 | 0 | 0 | 0 | 0 |

**ID:** I corresponds to values in the data for a generic health facility catchment area in the intervention group; C corresponds to values in the data for a generic health facility catchment area in the comparison group

**Model variables:**

- ***Y*:** Dependent variable. Corresponds to the malaria cases at the HFCA-level by month (t)
- ***T*:** Months since the start of the study. In this example, the intervention starts in month 3
- ***X***: 0 indicates pre-intervention period and 1 indicates post-intervention period
- ***XT***: Interaction term of X x T, which starts in the month immediately following the start of the intervention
- ***Z***: 0 indicates comparison group and 1 indicates intervention group
- ***ZT***: Interaction term of Z x T
- ***ZX***: Interaction term of Z x X
- ***ZXT***: Interaction term of Z x X x T, which starts in the month immediately following the start of the intervention
- **Covariates** included monthly number of outpatient visits to the health facilities, coverage of indoor residual spraying, temperature (with a 1- and 2-month lag), rainfall (with a 1- and 2-month lag), and enhanced vegetation index (with a 1- and 2-month lag).
